# Attenuation of advanced atherosclerosis and aortic aneurysm in hph-1/apoE DKO mice by folic acid and DHFR overexpression

**DOI:** 10.3389/fphys.2026.1864121

**Published:** 2026-06-30

**Authors:** Yixuan Zhang, Kin Lung Siu, Kai Huang, Yuanli Huang, Hua Cai

**Affiliations:** 1 Division of Molecular Medicine, Department of Anesthesiology and Perioperative Medicine, David Geffen School of Medicine, University of California, Los Angeles, Los Angeles, CA, United States; 2 Division of Cardiology, Department of Medicine, David Geffen School of Medicine, University of California, Los Angeles, Los Angeles, CA, United States

**Keywords:** aortic aneurysm, apoE null mice, atherosclerosis, dihydrofolate reductase (DHFR), eNOS uncoupling, folic acid (FA), hph-1 mice, hph-1/apoE double mutant mice

## Abstract

**Introduction:**

Atherosclerosis and aortic aneurysms often co-exist, attributed to common risk factors predisposing to development of these severe vascular disorders. The apoE null mice represent a classical model of atherosclerosis. It has been shown that endothelial nitric oxide synthase (eNOS) is modestly uncoupled in apoE null mice at baseline to contribute to oxidative stress.

**Methods:**

Notably, we have previously demonstrated marked activation of eNOS in its uncoupled state in angiotensin II (Ang II) infused hph-1 mice, which triggers formation of abdominal aortic aneurysm (AAA), representing the first evidence documenting a direct causal role of eNOS uncoupling in AAA formation that can be reversed by recoupling of eNOS with oral administration of folic acid (FA) or plasmid-mediated overexpression of dihydrofolate reductase (DHFR). In the present study, hph-1(hyperphenylalaninemia-1)/apoE double mutant mice were created in-house, and exposed to Ang II infusion (0.7 mg/kg/day) for 2 weeks alongside apoE null mice, during which formation of AAA was monitored by echo determination of abdominal aortic size on weekly basis and post-mortem inspection.

**Results:**

Indeed, although AAA formation was not significantly different between apoE and hph-1/apoE mice, there were significantly more atherosclerotic lesions formed in hph-1/apoE double mutant mice. These data indicate that AAA forms consequent to a certain extent of eNOS uncoupling activity. By contrast, worsened eNOS uncoupling activity is still associated with deteriorated atherogenesis in hph-1/apoE double mutant mice. In addition, oral FA administration or overexpression of DHFR substantially alleviated excessive atherosclerosis and AAA formation in apoE null mice and hph-1/apoE double mutant mice.

**Discussion:**

These findings seem to establish that targeting DHFR to fully restore eNOS function or to fully recouple eNOS into its native coupled state, can be employed as a novel and robust therapeutic strategy to alleviate advanced atherosclerosis.

## Introduction

Aortic aneurysm is a progressive and lethal vascular disease that is known as the second most predominant aortic disease next to atherosclerosis (Bossone and Eagle, 2021; Isselbacher et al., 2022; Zong et al., 2024). At the same time, atherosclerosis and aortic aneurysms often co-exist, at least in part attributed to the presence of common risk factors predisposing to the development of these severe vascular disorders, which can include dyslipidemia and hypertension. Of note, inflammation derived from oxidative stress represents a common mediator for both atherosclerosis and aortic aneurysm, as well as the development of hypertension (Cai and Harrison, 2000; Cai et al., 2003; Cai, 2005a; Cai, 2005b; Peshkova et al., 2016; Zhang et al., 2020). We and others have previously shown that uncoupling/dysfunction of endothelial nitric oxide synthase (eNOS) plays a driving role in both aortic aneurysm formation and atherogenesis (Cai et al., 1998; Laursen et al., 2001; Gao et al., 2012; Siu and Cai, 2014; Siu et al., 2014; Miao et al., 2015; Siu et al., 2017; Daiber et al., 2019; Li et al., 2019; Zhang et al., 2020; Huang et al., 2021a; Huang et al., 2021b; Huang et al., 2022; Zhang et al., 2022; Zong et al., 2024). Nonetheless, it has remained unclear whether augmented risk profiles attributed to the combination of more than one contributor [e.g., from both dyslipidemia in apolipoprotein E (apoE) null mice and eNOS dysfunction in hyperphenylalaninemia-1 (hph-1) mice] or to the heightened severity of vascular dysfunction (e.g., enhanced eNOS uncoupling activity) are detrimental in the disease processes, thus resulting in worsened aneurysm, atherosclerosis, or both. For example, it has remained interesting to explore whether additive eNOS uncoupling activities triggered by different etiologies would have synergistic effects in augmenting atherosclerosis and/or the formation of aortic aneurysms.

The apoE null mice represent a classical model of atherosclerosis. It has been shown that eNOS is modestly uncoupled in apoE null mice at baseline, contributing to atherogenesis especially under high-fat diet feeding (Laursen et al., 2001; Siu et al., 2014). We have previously documented marked activation of eNOS in its uncoupled state in angiotensin II (Ang II)-infused hph-1 mice, which drives the formation of abdominal aortic aneurysm (AAA), representing the first evidence establishing a direct causal role of eNOS uncoupling in AAA formation that can be reversed by recoupling of eNOS with oral administration of folic acid (FA) or plasmid-mediated overexpression of dihydrofolate reductase (DHFR) (Gao et al., 2012; Siu and Cai, 2014; Siu et al., 2014; Miao et al., 2015; Siu et al., 2017; Li et al., 2019; Zhang et al., 2020; Huang et al., 2021a; Huang et al., 2021b; Huang et al., 2022; Zhang et al., 2022). We have further shown that oral administration combining FA and Nifedipine can serve as a first-in-class oral medication fully alleviating AAA formation, via the restoration of DHFR protein abundance and activity to preserve eNOS/endothelial function (Huang et al., 2022). In addition, overexpression of transcriptional factor E2F1 to increase DHFR mRNA and protein levels is also robustly effective in reducing blood pressure via the preservation of functional/coupled eNOS activity (Li et al., 2019).

We have previously established a novel and more human-like model of AAA, by infusing Ang II into hph-1 mice. The aneurysm has a typical suprarenal localization, and it ruptures frequently and quickly as in human patients (Gao et al., 2012; Siu and Cai, 2014; Siu et al., 2014; Miao et al., 2015; Siu et al., 2017; Li et al., 2019; Zhang et al., 2020; Huang et al., 2021a; Huang et al., 2021b; Huang et al., 2022; Zhang et al., 2022). The other classical model of AAA is Ang II-infused apoE null mice, which requires 4 weeks of Ang II infusion at higher dosing (Daugherty and Cassis, 2004; Phie et al., 2021). The aneurysms do not rupture as much in the apoE null mice, although the abdominal aorta dilates more. Recoupling of eNOS is robustly effective in alleviating AAA formation in Ang II-infused apoE null mice as well (Siu et al., 2014). In the present study, we aimed to investigate whether hph-1/apoE double mutant mice innovatively created in-house, in which eNOS function would be further impaired in its uncoupled state, develop worsened atherosclerosis and/or deteriorated formation of AAA in response to Ang II infusion.

## Methods

### Mouse models

The hph-1 mice have been bred in-house continuously for aneurysm studies (Gao et al., 2012; Miao et al., 2015; Huang et al., 2021a). The apoE null mice founders were obtained from Jackson Labs and bred in-house. The hph-1/apoE double mutant mice were generated by crossing hph-1 mice with apoE null mice until both backgrounds are in homogeneous states. The hph-1 mice were previously backcrossed over 10 generations into C57BL/6 background, so that the hph-1/apoE double mutant mice are also in the C57BL/6 background. The apoE null alone mice were used as the comparative control group for the hph-1/apoE double mutant mice, since the experimental design was to examine whether the hph-1 background exerts additional impacts on disease processes under the apoE null baseline.

### Ang II infusion into hph-1/apoE double mutant and apoE null mice

For observation of AAA formation and atherogenesis, 6-month-old male hph-1/apoE double mutant mice and apoE null mice were infused with Ang II (0.7 mg/kg/day) for 2 weeks as we previously established for hph-1 mice (Gao et al., 2012; Miao et al., 2015; Huang et al., 2021a). Of note, the dosing used for Ang II infusion into hph-1/apoE double mutant mice was aligned with dosing employed for Ang II-infused hph-1 mice, since the experiments were designed to primarily dissect out potential impacts on disease processes of hph-1 background. During the treatment period, mice were monitored of abdominal aortic expansion using echo on a weekly basis as described below and previously published (Gao et al., 2012; Siu and Cai, 2014; Siu et al., 2014; Miao et al., 2015; Siu et al., 2017; Li et al., 2019; Zhang et al., 2020; Huang et al., 2021a; Huang et al., 2021b; Huang et al., 2022; Zhang et al., 2022). Upon harvest, mouse aortas were isolated for post-mortem inspection to observe AAA formation.

### Echo monitoring of abdominal aortic expansion

The ultrasound monitoring of abdominal aortic expansion was performed as we have previously published (Gao et al., 2012; Siu and Cai, 2014; Siu et al., 2014; Miao et al., 2015; Siu et al., 2017; Li et al., 2019; Zhang et al., 2020; Huang et al., 2021a; Huang et al., 2021b; Huang et al., 2022; Zhang et al., 2022). In brief, animals were anesthetized with isoflurane and placed on a temperature-controlled table, which also measures ECG for heart rate. Isoflurane levels were adjusted throughout the experiment to maintain heart rate between 400 and 500 bpm while keeping the animal sufficiently anesthetized. Hair was removed from the abdomen using a hair removal cream, and preheated ultrasound transmission gel was applied onto the abdomen area. An ultrasound probe (Velvo 770, Visualsonics) was placed on the gel to visualize aorta transversely. The aorta was identified using Doppler measurement for the presence of pulsatile flow. Consistent localization of image acquisition was insured by visualizing the aorta immediately superior to the branch of the left renal artery in all of the animals. Images were recorded and saved onto a an offline desktop computer for offline area analysis.

### Oil Red O staining for assessing atherosclerotic lesions

A stock of 0.5% (w/v) of Oil Red O (ORO, #O0625, MilliporeSigma, Burlington, MA) was prepared in isopropanol. The working solution of 0.3% ORO was prepared by diluting the stock solution with deionized water and filtration with a 0.2-μm filter. Cleaned mouse aortas were rinsed in phosphate-buffered saline (PBS) containing 0.5% Triton X-100 and stained with ORO working solution for 30 min at room temperature. Then, the aortas were destained with isopropanol and washed in distilled water for 20 min. Images were captured using Leica Microscope M60 with Leica Microsystems Digital Camera EC3. Lesion areas and areas of whole aortas were analyzed by NIH Image J Software. The percentage of lesion areas was calculated as 100% * lesion area/area of whole aorta.

### Statistical analysis

Data are presented as mean ± SEM. GraphPad software was used for statistical analysis. For AAA incidence among different groups, chi-square analysis was used. For comparison of means among multiple groups, one-way analysis of variance (ANOVA) was employed and followed by *post-hoc* analysis. *p* < 0.05 is considered to indicate statistically significant difference.

## Results

**Figure 1:**
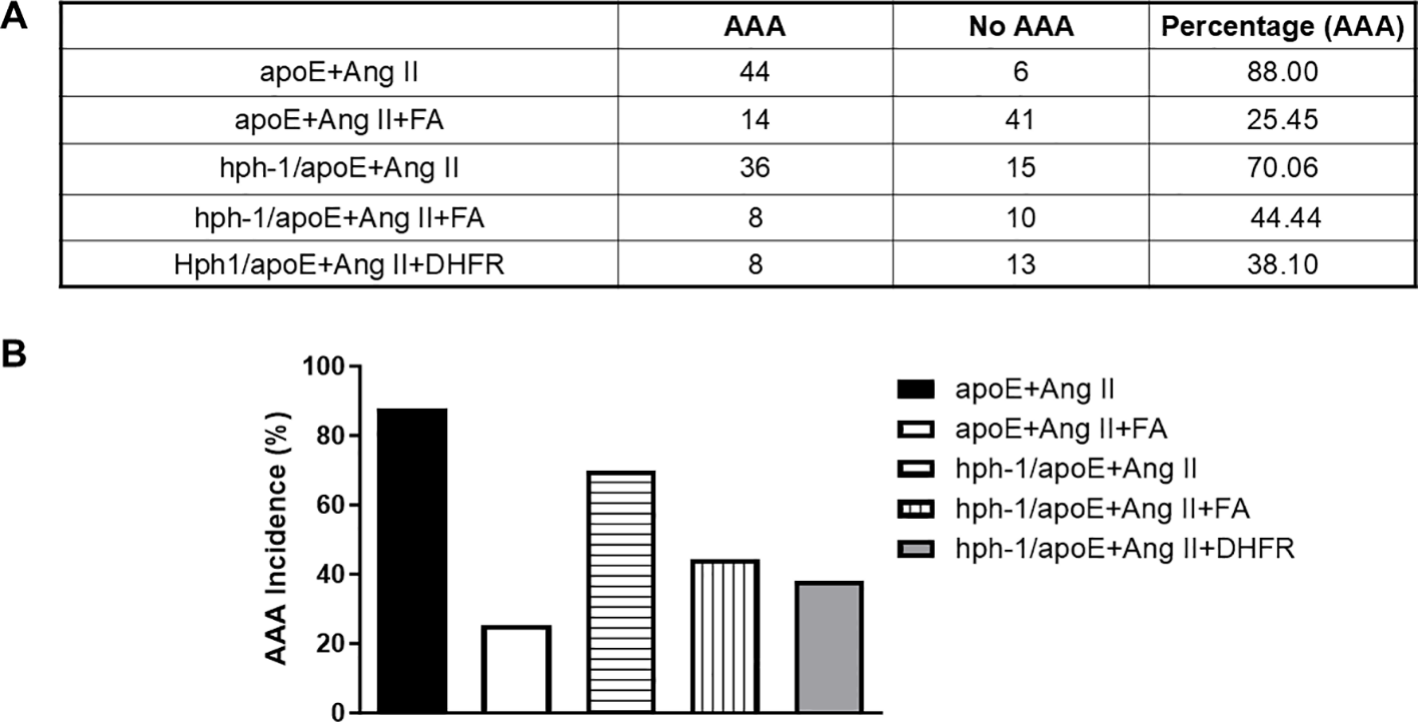
Oral folic acid (FA) administration and dihydrofolate reductase (DHFR) overexpression attenuated incidence of abdominal aortic aneurysm (AAA) formation in hph-1/apoE double mutant mice. The hph-1/apoE double mutant mice and apoE null mice (all at 6 months old) were infused with angiotensin II (Ang II, 0.7 mg/kg/day) for 2 weeks. Some mice were orally treated with folic acid using customized chew (15 mg/kg/day) or subjected to plasmid-based overexpression of DHFR as we have previously shown. Mice were dissected for post-mortem inspection to determine incidence of AAA. **(A, B)** Incidence rates of AAA in different experimental groups. Data indicate that the oral FA administration or overexpression of DHFR robustly attenuated AAA formation in both hph-1/apoE double mutant mice and apoE null alone mice exposed to Ang II. *n* = 60, 55, 51, 18, and 22 for apoE+Ang II, apoE+Ang II+FA, hph-1/apoE+Ang II, hph-1/apoE+Ang II+FA, and hph-1/apoE+Ang II+DHFR groups, respectively. Chi-square analysis was used for statistical analysis. *p* < 0.0001 for apoE+Ang II+FA vs. apoE+Ang II; *p* = 0.047 for hph-1/apoE+Ang II+FA vs. hph-1/apoE+Ang II; *p* = 0.010 for hph-1/apoE+Ang II+DHFR vs. hph-1/apoE+Ang II.

Male hph-1/apoE double mutant mice were created in-house and exposed to Ang II infusion (0.7 mg/kg/day) for 2 weeks alongside apoE null mice both at 6 months old, during which the formation of AAA was monitored by echo determination of abdominal aortic size on a weekly basis as we have previously published (Gao et al., 2012; Siu and Cai, 2014; Siu et al., 2014; Miao et al., 2015; Siu et al., 2017; Li et al., 2019; Zhang et al., 2020; Huang et al., 2021a; Huang et al., 2021b; Huang et al., 2022; Zhang et al., 2022). At the end of 2 weeks, mice were harvested for post-mortem inspection to determine AAA incidence, while ORO staining was used to assess atherosclerotic lesions. As shown in **Figures 1A, B**, the incidence of AAA was 88.00% in apoE null mice and 70.06% in hph-1/apoE double mutant mice, respectively, which was not significantly different. Oral administration with FA was able to attenuate AAA incidence rate to 25.45% in apoE null mice, similar to our previous observations (Siu et al., 2014). Likewise, but innovatively, FA diet or DHFR overexpression was equally effective in significantly diminishing AAA incidence in hph-1/apoE double mutant mice to 44.44% (*p* = 0.047) and 38.10% (*p* = 0.010), respectively (**Figures 1A, B**, by chi-square analysis).

**Figure 2:**
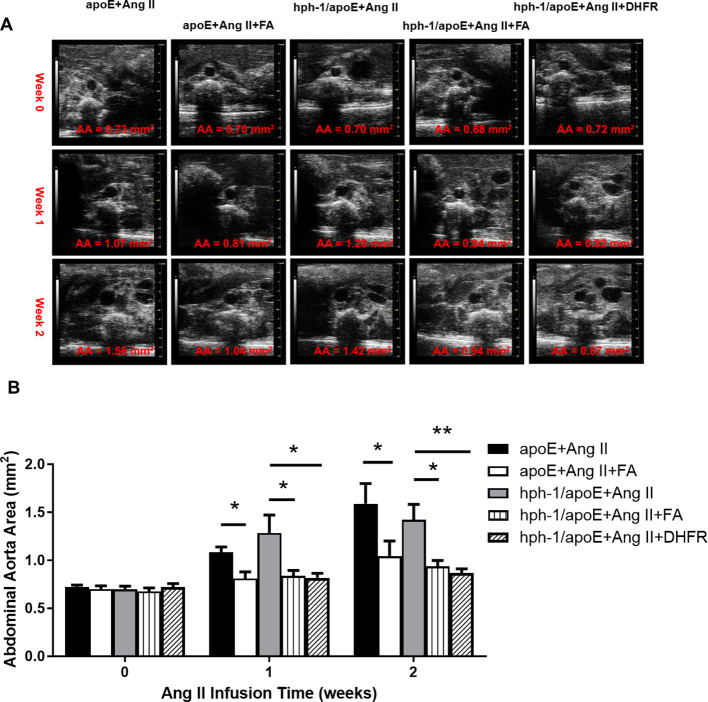
Oral folic acid (FA) administration and dihydrofolate reductase (DHFR) overexpression attenuated echo-defined expansion of abdominal aorta in hph-1/apoE double mutant mice. The hph-1/apoE double mutant mice and apoE null mice (all at 6 months old) were infused with angiotensin II (Ang II, 0.7 mg/kg/day) for 2 weeks. Some mice were orally treated with folic acid using customized chew (15 mg/kg/day) or subjected to plasmid-based overexpression of DHFR as we have previously shown. The size of abdominal aorta was measured by echo on a weekly basis as we have previously published. **(A)** Representative echo images of abdominal aortic expansion in different experimental groups. **(B)** Grouped quantitative data of abdominal aortic expansion in different experimental groups. Data indicate that oral FA administration or overexpression of DHFR robustly attenuated abdominal aortic expansion in both hph-1/apoE double mutant mice and apoE null alone mice exposed to Ang II. *n* = 7–9, *p<0.05, **p<0.01 for the marked comparisons by one-way ANOVA analysis.

In addition to post-mortem inspection, AAA formation was also assessed by echo defined abdominal aortic expansion. There seemed to be a tendency of deteriorated response in hph-1/apoE double mutant mice at week 1, as shown in **Figures 2A, B**. Of note, **Figures 2A, B**, illustrate representative echo images and grouped quantitative data respectively from various experimental groups. This trend of deteriorated AAA formation was no longer obvious by week 2 (**Figures 2A, B**). Similar to AAA incidence rate by post-mortem inspection, oral FA administration or DHFR overexpression was able to abrogate echo-defined abdominal aortic expansion/AAA formation in hph-1/apoE double mutant mice (**Figures 2A, B**). As described in the figure legend, *p* < 0.05 indicates significant efficacies of FA and DHFR overexpression in alleviating AAA formation in both hph-1/apoE double mutant mice and apoE null mice.

**Figure 3:**
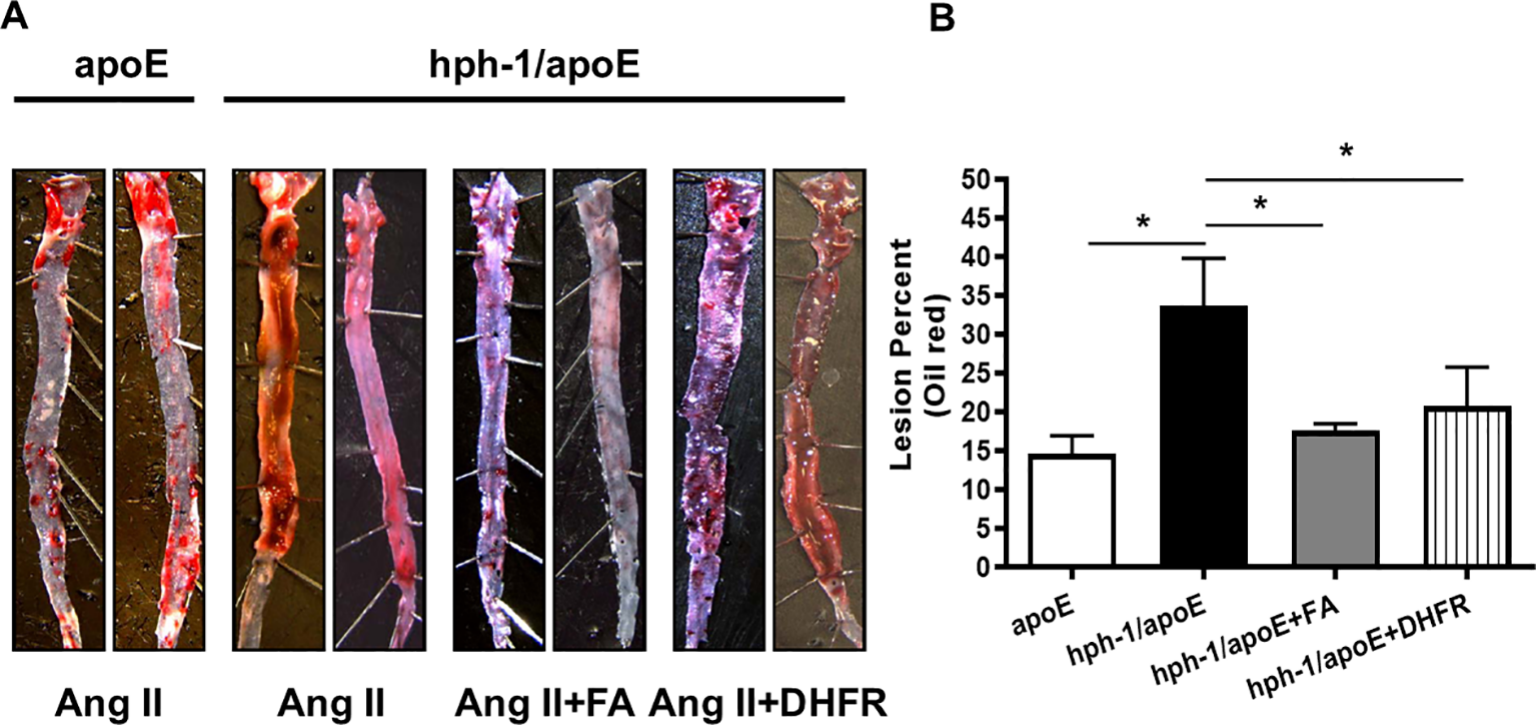
Oral folic acid (FA) administration and dihydrofolate reductase (DHFR) overexpression attenuated advanced atherosclerotic lesion formation in hph-1/apoE double mutant mice. The hph-1/apoE double mutant mice and apoE null mice (all at 6 months old) were infused with angiotensin II (Ang II, 0.7 mg/kg/day) for 2 weeks. Some mice were orally treated with folic acid using customized chew (15 mg/kg/day) or subjected to plasmid-based overexpression of DHFR as we have previously shown. The atherosclerotic lesions were assessed by Oil Red O staining. **(A)** Representative images of Oil Red O staining in different experimental groups. **(B)** Grouped quantitative data of Oil Red O staining in different experimental groups. Data indicate markedly more advanced atherosclerotic lesion formation in hph-1/apoE double mutant mice compared to the apoE null alone mine. Oral FA administration or overexpression of DHFR robustly attenuated advanced formation of the atherosclerotic lesions in hph-1/apoE double mutant mice, while it was equally effective in apoE null alone mice. *n* = 7–9, *p<0.05 for the marked comparisons by one-way ANOVA analysis.

Strikingly, there was a significant further increase in atherosclerotic lesion formation in hph-1/apoE double mutant mice compared to apoE null mice, as assessed by ORO staining (**Figures 3A, B**), which was innovatively and near fully alleviated by oral FA administration or DHFR overexpression to recouple eNOS or restore eNOS function. Data in **Figures 3A, B**, illustrate representative ORO images and quantitative grouped data from different experimental groups. As described in the figure legend, *p* < 0.05 indicates a significant increase in lesion formation in hph-1/apoE double mutant mice compared to apoE null alone mice, and marked efficacies of FA and DHFR overexpression in alleviating lesion formation in hph-1/apoE double mutant mice. These data seem to indicate that, synergistically, further augmented eNOS uncoupling activity in hph-1/apoE double mutant mice is able to drive deteriorated and excessive atherogenesis. Hence, targeting DHFR pathway to preserve eNOS function could accordingly serve as a novel and robust therapeutic option for advanced atherosclerosis.

## Discussion

Although an important mediator role of eNOS uncoupling has been implicated in the pathogenesis of vascular diseases such as hypertension and atherosclerosis (Li et al., 2015; Daiber et al., 2019; Zhang et al., 2020), it has remained unclear whether the degree or extent of eNOS uncoupling activity determines or dictates the severity of the disease. In the present study, we used a novel genetic model of hph-1/apoE double mutant mice generated in-house for the first time, in which eNOS uncoupling is anticipated to have worsened synergistically following additive loss in eNOS cofactor tetrahydrobiopterin (H_4_B) from an hph-1 background (mutant in H_4_B synthetic enzyme GTPCHI) (McDonald and Bode, 1988) and an apoE null background (H_4_B oxidation due to elevated lipid levels/dyslipidemia) (Laursen et al., 2001; Siu and Cai, 2014; Siu et al., 2014; Siu et al., 2017), to examine whether the development of AAA and atherosclerosis is also correspondingly augmented. Indeed, although AAA formation was not significantly different between apoE null and hph-1/apoE double mutant mice under Ang II infusion, there was significantly more atherosclerotic lesions formed in hph-1/apoE double mutant mice. These compelling data seem to indicate that AAA formation might be consequent to a certain threshold of eNOS uncoupling activity, so that further uncoupling of eNOS is no longer pathologically significant. Of note, Ang II infusion into hph-1 mice induced tripled eNOS uncoupling activity compared to Ang II-infused wild-type mice (Gao et al., 2009; Gao et al., 2012).

By contrast, worsened eNOS uncoupling activity is still associated with deteriorated and more advanced atherogenesis. This was previously untested and unexplored, and it was previously unclear whether deteriorated eNOS dysfunction is proportional to degree of atherosclerosis (Li et al., 2015; Daiber et al., 2019; Zhang et al., 2020). Of note, our new findings in the present study indicate that oral FA administration and DHFR expression almost fully alleviated atherosclerosis and AAA formation in both apoE null and hph-1/apoE double mutant mice (**Figures 3A, B**). These observations seem to also indicate that targeting DHFR to restore eNOS function or fully recouple eNOS into its native state represents a novel and robust therapeutic strategy to treat advanced atherosclerosis. When eNOS is uncoupled due to deficiency in DHFR, levels of tetrahydrobiopterin (H_4_B) is reduced as a causal factor proceeding eNOS uncoupling. We have previously characterized decreased H_4_B levels in Ang II-infused hph-1 mice (Gao et al., 2012; Siu et al., 2017; Li et al., 2019) and Ang II-infused apoE null mice (Siu et al., 2014; Huang et al., 2022). It is foreseeable that H_4_B levels are significantly reduced in Ang II-infused hph-1/apoE null mice as well, although repeat measures in this strain might not be necessary when our primary goal is to examine any significant phenotypical differences among the different genetic strains.

In the present study, hph-1/apoE double mutant mice was compared to one model of atherosclerosis in apoE null mice, while apoE null mice was used as a control in this setting to offer the same background of dyslipidemia. Of note, in another model of atherosclerosis, LDL receptor (LDLR) null mice, there is also baseline phenotype of eNOS uncoupling (Musicki et al., 2010; Ning et al., 2021). Hence, comparison of hph-1/LDLR double mutant mice to LDLR null mice might provide additional confirmation to the innovative concept that synergistically worsened eNOS uncoupling activity drives more advanced atherogenesis. Another consideration underlies experimental design is that lower levels of Ang II (0.7 mg/kg/day) were used in the hph-1/apoE double mutant mice compared to what is usually used in apoE null mice for induction of AAA formation, which might have confounded the responses in aortic aneurysm formation. Nonetheless, we used the dosing that is equivalent to the established AAA model of Ang II infusion into hph-1 mice, since our primary goals were to examine additional inputs on disease processes from the hph-1 background.

Our current study primarily focuses on pathophysiological differentiation in phenotypes of atherosclerosis and aortic aneurysm in Ang II-infused hph-1/apoE double mutant mice compared to apoE null mice. Although additional measurements of blood pressure and lipid levels can be helpful, we have previously characterized these factors in Ang II-infused hph-1 mice to show similarly elevated blood pressure compared to wild-type control mice by days 5–6 post Ang II infusion, and declined blood pressure when hph-1 mice started to die of severely ruptured aneurysms (Gao et al., 2012). It is anticipated that lipid levels will be elevated in hph-1/apoE null mice similarly to what is observed in apoE null mice. We did not observe any changes in lipid levels in mice of the hph-1 background. There has also not been any direct evidence that further uncoupling of eNOS can cause regulation of lipid levels to deteriorate dyslipidemia. Exaggerated atherogenesis in Ang II-infused hph-1/apoE null mice is, however, considered attributed primarily to more severe endothelial dysfunction, triggered by more severe uncoupling activity of eNOS.

Of note, the hph-1 mutation impairs GTPCHI-dependent H_4_B synthesis to also affect hepatic H_4_B levels, which then reduces phenylalanine hydroxylase activity, leading to elevated plasma phenylalanine (Lam et al., 2007). Whereas previous studies and our work seem to suggest that vascular pathologies in hph-1 mice are primarily attributed to aortic H_4_B deficiency and consequent eNOS uncoupling activity, measurement of circulating phenylalanine levels warrants further investigation. It will help to dissect if any phenylalanine-driven oxidative metabolites are involved in vascular inflammation and endothelial dysfunction. Other factors to consider are traditional risk factors for AAA formation, incorporation of which into the hph-1/apoE null double mutant background might further increase or deteriorate atherogenesis and/or development of AAA. Of note, cigarette smoking and male gender are the most important risk factors for AAA formation in human patients (Aune et al., 2018; Bossone and Eagle, 2021). Nonetheless, the potential impacts on AAA formation of the emerging and increasing use of e-Cigarettes (e-Cig) have not been fully investigated (Cai and Wang, 2017; Cai et al., 2020; Liu et al., 2022). The latest studies seem to have demonstrated effects on AAA formation of e-Cig vapes in both Ang II-infused apoE null mice and the elastase infusion model (Mulorz et al., 2023). Another emerging risk factor is COVID-19 infection, which has been known recently to have major and chronic impacts on the cardiovascular system (Cai, 2020; Youn et al., 2021; Youn et al., 2022; Zhou et al., 2024), including deterioration of aortic aneurysms (Cao et al., 2024; Huang et al., 2024). Integration of classical and emerging risk profiles into future experimental designs may dissect out more details of multifactorial determining algorism that all lead to the central and triggering event of eNOS/endothelial dysfunction.

Taken together, our data for the first time indicate that whereas a certain degree of eNOS uncoupling is sufficient to trigger aortic aneurysm formation, augmented eNOS uncoupling activity drives more severe atherosclerosis in hph-1/apoE double mutant mice. Targeting DHFR with overexpressing construct or FA diet proves to be innovatively effective in markedly alleviating the advanced formation of atherosclerotic lesions. Therefore, targeting DHFR to preserve eNOS/endothelial function might represent an innovative and significant new therapeutic option for the treatment of advanced atherosclerosis.

## Data Availability

The original contributions presented in the study are included in the article/supplementary material, further inquiries can be directed to the corresponding author/s.
